# Maternal Cognitive Impairment Associated with Gestational Diabetes Mellitus—A Review of Potential Contributing Mechanisms

**DOI:** 10.3390/ijms19123894

**Published:** 2018-12-05

**Authors:** Cini Mathew John, Nur Intan Saidaah Mohamed Yusof, Siti Hajar Abdul Aziz, Fazlin Mohd Fauzi

**Affiliations:** 1 Department of Pharmacology and Chemistry, Faculty of Pharmacy, Universiti Teknologi MARA, Bandar Puncak Alam 42300, Selangor Darul Ehsan, Malaysia; cini.john@ucalgary.ca (C.M.J.); ndynise@gmail.com (N.I.S.M.Y.); sitihajaraziz92@gmail.com (S.H.A.A.); 2 Department of Physiology and Pharmacology, Faculty of Medicine, University of Calgary, 3330 Hospital Dr. NW, Calgary, AB T2N 4N1, Canada

**Keywords:** gestational diabetes mellitus, cognitive impairment, neuroinflammation, insulin resistance, oxidative stress

## Abstract

Gestational diabetes mellitus (GDM) carries many risks, where high blood pressure, preeclampsia and future type II diabetes are widely acknowledged, but less focus has been placed on its effect on cognitive function. Although the multifactorial pathogenesis of maternal cognitive impairment is not completely understood, it shares several features with type 2 diabetes mellitus (T2DM). In this review, we discuss some key pathophysiologies of GDM that may lead to cognitive impairment, specifically hyperglycemia, insulin resistance, oxidative stress, and neuroinflammation. We explain how these incidents: (i) impair the insulin-signaling pathway and/or (ii) lead to cognitive impairment through hyperphosphorylation of τ protein, overexpression of amyloid-β and/or activation of microglia. The aforementioned pathologies impair the insulin-signaling pathway primarily through serine phosphorylation of insulin receptor substances (IRS). This then leads to the inactivation of the phosphatidylinositol 3-kinase/Protein kinase B (PI3K/AKT) signaling cascade, which is responsible for maintaining brain homeostasis and normal cognitive functioning. PI3K/AKT is crucial in maintaining normal cognitive function through the inactivation of glycogen synthase kinase 3β (GSκ3β), which hyperphosphorylates τ protein and releases pro-inflammatory cytokines that are neurotoxic. Several biomarkers were also highlighted as potential biomarkers of GDM-related cognitive impairment such as AGEs, serine-phosphorylated IRS-1 and inflammatory markers such as tumor necrosis factor α (TNF-α), high-sensitivity C-reactive protein (hs-CRP), leptin, interleukin 1β (IL-1β), and IL-6. Although GDM is a transient disease, its complications may be long-term, and hence increased mechanistic knowledge of the molecular changes contributing to cognitive impairment may provide important clues for interventional strategies.

## 1. Introduction

Gestational diabetes mellitus (GDM) is defined by the American Diabetes Association as occurring in pregnant women who were not diagnosed with diabetes before pregnancy but have high blood glucose levels during pregnancy, usually around the 24th week [1]. The overall incidence of GDM is dramatically increasing, according to the International Diabetes Federation, who estimated that 200 million women suffered from GDM in 2015. GDM is projected to increase to 300 million in 2030 [2]; furthermore, between 10% and 25% of pregnancies suffer from GDM according to World Health Organization (WHO) reports in 2016 [3]. Women who develop GDM present a metabolic condition similar to that of type 2 diabetes mellitus (T2DM), characterized by insulin resistance coupled with defective insulin signaling [4,5,6]. Due to the similar pathophysiological mechanisms between T2DM and GDM, there will be common biomarkers that contribute to the complications of both diseases [7,8]. One such complication is cognitive decline, where metabolic syndrome is an established risk factor, which was first explained in the Rotterdam Study. The Rotterdam Study is a prospective, population-based cohort study, which investigated factors that determined the presence of neurological diseases and diabetes mellitus [9]. Results from this study showed that about 8.8% of diabetes cases present cognitive impairment. Additionally, diabetes has been established as a risk factor of vascular cognitive impairment [10]. Several preclinical and clinical studies have suggested that cognitive impairment in T2DM can been correlated with pathophysiology that includes hyperglycemia, insulin resistance, inflammation, vascular changes, and amyloid lesions [11,12,13,14,15,16,17]; accordingly, GDM clearly shares some features of the multifactorial pathogenesis seen in T2DM associated cognitive dysfunction. Reduction in insulin sensitivity and impaired insulin secretion are similar in both cases. However, hyperglycemia found in GDM is generally transitory and blood glucose level will normally return to normal after delivery [5].

Cognitive decline in GDM patients was first reported by Keskin et al. [18] where the association between cognitive function and metabolic status in 44 patients with GDM and 45 normal pregnant women were evaluated. GDM women aged above 30 years with a high body mass index (BMI) and glucose level were subjected to several cognitive function tests such as Montreal Cognitive Assessment, Symbol Digit Modalities Test and Spatial Recall Test, and were compared to non-diabetic women [18]. Based on the 30-point test in Montreal Cognitive Assessment, which evaluates mild cognitive impairment, GDM women scored 21 points and healthy pregnant women scored 24 points. The Symbol Digit Modalities requires women to pair abstract symbols and specific numbers within 90 s. GDM women showed poor speed of mental activity and attention, scoring five points lower than normal healthy pregnant women. Moreover, in the Spatial Recall Test and a visual memory test, women with GDM scored 4.5 points lower than normal pregnant women, with a score of 5.4 points [18].

Similarly, the brain development of the offspring of GDM women is also suspected to be affected from the prenatal stage. In a meta-analysis study, which examined the link between maternal diabetes and cognitive performance in offspring of GDM mothers, data were extracted from different electronic databases such as SciFinder, Scopus, The Cochrane Library and ClinicalTrial.gov. Infants from a GDM mother showed lower scores of mental and psychomotor development when compared to healthy pregnant women, with a mean standardized difference of −0.41 for mental development and −0.31 for psychomotor development [19]. Another study conducted by Linder et al. [20] showed that the postprandial auditory response of fetuses of GDM mothers is slower than the fetuses of normal glucose-tolerant (NGT) mothers. The auditory response of the fetus was recorded with a fetal magnetoencephalographic device after pregnant participants had undergone an oral glucose tolerance test (OGTT, 75 g). Sixty minutes after glucose ingestion, the auditory response latency of the GDM group was longer than in the NGT group (296 ± 82 ms vs. 206 ± 74 ms, *p* = 0.001), suggesting that the neural activity of fetuses of GDM mothers is slower than fetuses of NGT mothers [20].

**Figure 1:**
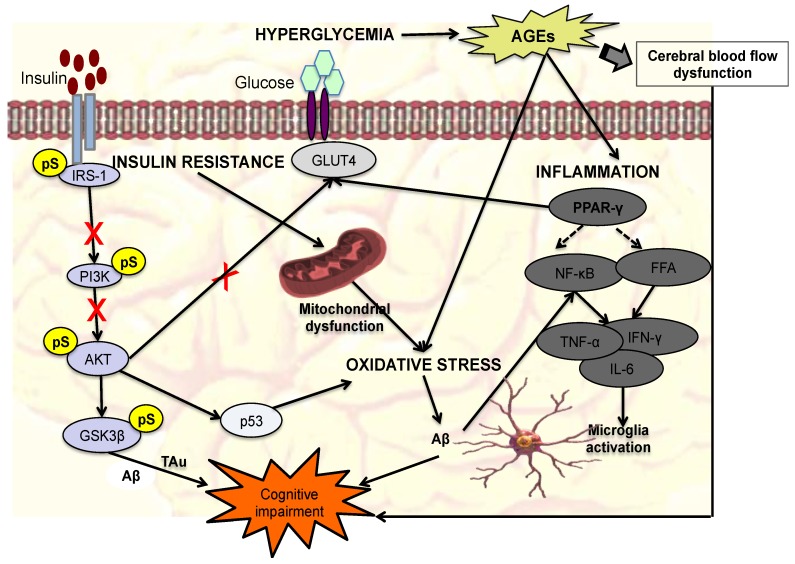
**Summary of the proposed mechanisms of maternal cognitive impairment associated with gestational diabetes mellitus (GDM).** The red ‘X’ and dashed arrows indicate inhibition and negative regulation respectively. Four mechanisms of GDM-induced cognitive impairment are covered here, which are hyperglycemia, insulin resistance, oxidative stress, and inflammation. In hyperglycemic condition, advanced glycation end products (AGEs) are overproduced, leading to oxidative stress, inflammation, and dysfunction of cerebral flow. Dysfunction of cerebral flow can lead to cognitive dysfunction through disruption of blood-brain barrier (BBB) transport mechanism that transport important molecules such as choline into the brain and clears unwanted molecules such as amyloid-β (Aβ) protein. In cases of insulin resistance, the insulin-signaling pathway is inactivated primarily through serine phosphorylation of insulin receptor substance (IRS-1). This inactivates the phosphatidylinositol 3-kinase/protein kinase B (PI3K/AKT) signaling cascade, which blocks the translocation of glucose transporter 4 (GLUT4), decreasing glucose uptake. Additionally, glycogen synthase kinase 3β (GSK3β) is also activated causing the hyperphosphorylation of τ proteins and overexpression of Aβ proteins. Instances such as overexpression of AGEs, insulin resistance and mitochondrial dysfunction can lead to oxidative stress, which cause the overexpression of Aβ. Free fatty acid (FFA) triggers activation of the microglia via nuclear factor kappa β (NF-κB) signaling production of pro-inflammatory cytokines (tumor necrosis factor α; TNF-α, interleukin 1β; IL-1β, interleukin 6; IL-6) additionally, Aβ induces the production of Nuclear factor-kappa β; NF-κβ, which also releases pro-inflammatory cytokines.

It is clear that GDM affects the cognitive function of not just the mother but also the offspring; however, the effect on the offspring is studied more than that on the mothers. Given that the genesis of GDM-induced cognitive impairment begins in the mother and is passed onto their offspring, it is important to study the possible markers for GDM-associated cognitive decline and its pathophysiology. Unfortunately, mechanistic study on this is severely lacking. In this review, we looked at several pathophysiologies of GDM, T2DM, and other neurocognitive diseases which may shed more light on the mechanism of GDM-associated cognitive function. One of the findings from Genetic Wide Association Studies (GWAS) was that several different diseases are interconnected and may share several genes [21]. Hence, it is possible to gain insight of a disease by studying other diseases with which it may share similarities, such as on a genotypic and/or phenotypic level [21,22,23]. In addition, given the overlap between genes associated with both GDM and T2DM, and the observation that GDM mothers are at an increased risk for the development of T2DM [24], cognitive impairment associated with GDM might be long-term [25]. This further highlights the importance of understanding the pathophysiology of GDM-associated cognitive function in mothers. In this review, we will examine incidents of GDM that are potentially linked to maternal cognitive impairment viz. hyperglycemia, insulin resistance, oxidative stress and neuroinflammation. A summary of each of the markers, changes, and outcomes of each of the pathophysiologies can also be found in Table 1, and a figurative summary can be found in Figure 1.

## 2. Pathophysiology of GDM That Can Lead to Cognitive Impairment

### 2.1. Hyperglycemia

Hyperglycemia is the hallmark of type 1 diabetes mellitus (T1DM), T2DM, and GDM, resulting from a decline of insulin supply that is insufficient to meet tissue demands for normal blood glucose regulation and/or insensitivity of insulin towards insulin receptors [4]. Given that hyperglycemia is the common denominator among the three types of diabetes, it stands to reason that hyperglycemia may lead to cognitive impairment based on several studies [12]. Indeed, reports have demonstrated the positive correlation between glycated hemoglobin (HbA1c), a measure of blood sugar level, and cognitive decline. A study by Zeng et al. [65] looked at possible association between HbA1c levels and long-term cognitive decline where a 1 mmol/mol increase of HbA1c was significantly correlated with an increase rate of decline in tests including memory, executive function and global cognitive task [65]. Similar results were also observed in Gupta, et al. [66], although the study involves fewer participants than the former and were not conducted over a long period of time. 

One possible way that hyperglycemia induces cognitive impairment is through the production of advanced glycation end products (AGEs) (see Figure 1) [26]. Several studies have demonstrated the positive correlation between AGEs and cognitive impairment, leading to the suggestion that AGEs are a biomarker of cognitive impairment in diabetic conditions. One such study is by Spauwen et al. [26], which is a part of the Maastricht Study, a population-based study involving 215 patients with T2DM. In this study, the association between AGEs, obtained through non-invasive skin autofluorescence, and cognitive function of the patients were analyzed. Cognitive function such as verbal memory, global cognitive functioning, response inhibition and information processing speed were assessed. The study showed that AGEs level was positively correlated with worse delayed-word recall and response inhibition, leading to the conclusion that AGEs level is associated with poor cognitive performance [26]. Similar findings were also observed in Wang et al. [67] where soluble receptors of advanced glycation end products (sRAGE) and AGE-peptide (AGE-P) levels were measured in T2DM patients with mild cognitive impairment (MCI). In addition, their performance on several neuropsychological tests was also evaluated. Compared to the control, the MCI group showed a lower sRAGE level (0.87 vs. 1.05 ng/mL) and higher AGE-P level (3.55 vs. 2.71 U/mL) [67]. sRAGE was negatively correlated with TMT-B score, a test for early mild cognitive impairment, and AGE-P was negatively correlated with several tests assessing different cognitive domain [67]. AGEs are formed through non-enzymatic reactions between reduced sugars (usually glucose) and amino acids contained in proteins [68]. This process takes place under oxidative stress and/or hyperglycemic conditions, and produces Schiff base, from the complex formed between glucose and protein. The Schiff base is reversible but if the reaction continues, it undergoes a rearrangement involving a non-enzymatic glycosylation forming what is called an Amadori product. Amadori products are stable but less reversible compared to Schiff bases. After several weeks, the Amadori products stabilize and reach equilibrium. They will undergo further changes, ultimately transforming to the irreversible AGEs [69]. The cross-linking and glycation of proteins (such as collagen and amyloid) to produce AGEs can impair their function and structural integrity. Furthermore, the accumulation of AGEs, and its activation via its receptor RAGEs, evokes several reactions such as oxidative stress, inflammation, and fibrotic and thrombotic reactions [67]. All of these can lead to cognitive impairment. Regarding oxidative stress and inflammation, these will be explained in separate sections. 

Cognitive impairment and other central nervous system (CNS)-related dysfunction have been linked to the dysfunction of the neurovascular unit and regulation of cerebral blood flow [27,28]. The effect of hyperglycemia on the vascular system has been well documented where hyperglycemia induces abnormal proliferation of endothelial cells, thickening of capillary walls, lowering of perfusion rates and increase in vascular permeability [27]. These effects have been found to affect the integrity of the blood–brain barrier (BBB) and its transport function [27]. The BBB is the gatekeeper of the brain, where it is responsible for the maintenance of brain homeostasis by transporting essential molecules in and out of the CNS in accordance with the metabolic needs of the brain and protects the CNS from harmful substances. The BBB transport mechanism is responsible for transporting molecules such as choline across the BBB. Choline is a precursor of acetylcholine, a neurotransmitter that is responsible for functions such as memory, where lack of acetylcholine is known to be one of the causes of Alzheimer’s disease (AD). Vascular damage of the BBB can hinder the transport of choline, depriving the brain of an important neurotransmitter and affecting the homeostasis of the brain. Additionally, vascular damage can also reduce the clearance of amyloid-β (Aβ) protein, which then promotes tau (τ) phosphorylation causing cognitive impairment and disrupting the brain homeostasis. These chemicals can induce damage to the CNS through oxidative stress and inflammation. The BBB also protects the CNS from harmful substances such as free radicals and pro-inflammatory cytokines. In cases of damage to the BBB, these chemicals can easily infiltrate the CNS [29,30].

### 2.2. Insulin Resistance

Insulin resistance is another mechanism that can possibly contribute to the neuropathology of cognitive impairment in GDM, given the substantial amount of studies that have already been published linking cognitive dysfunction to insulin resistance [70,71,72,73]. In GDM, changes in hormones and increased adipose tissue are observed [74]. The placenta, which produces several hormones e.g., human placental lactogen, estrogen and progesterone, can cause insulin resistance by reducing the expression of glucose transporter-4 (GLUT4) and causing the dysfunction of β-cell adaptive response for enhancing insulin production. In addition, the increase in adipose tissue due to high intake of food to provide energy for the mother and for the development and growth of the fetus results in high production of adipocytokines. Adipocytokines are adipocyte hormones that are involved in the regulation of metabolism and insulin resistance such as TNF-α, adipokines, and leptin [74].

The insulin-signaling pathway is activated when insulin binds to an insulin receptor (IR), leading to the recruitment of adapter proteins such as insulin receptor substances (IRS) through the phosphorylation of its tyrosine residue (see Figure 1). In response, three major signaling pathways will be triggered, which are mitogen activated protein (MAP) kinase, casitas b-lineage lymphoma/Cbl-associated protein (Cbl/CAP), and phosphatidylinositol 3-kinase (PI3K) pathways. The PI3K cascade activates the AKT/PKB [47,75,76,77], causing the translocation of GLUT4 to the plasma membrane, increasing glucose uptake [31]. The insulin-signaling pathway can be impaired through two mechanisms, where (i) downstream components of the pathway (mTOR/S6K, MAPK, PKC) inhibit the upstream components, similar to the negative feedback mechanism, and (ii) inhibition by molecules (GSK3β, IKKβ, JNK, mPLK, AMPK) from other pathways. In the latter, this is achieved primarily through the serine phosphorylation of IRS, blocking further signal propagation. Serine-phosphorylated IRS has been suggested to be a biomarker of T2DM, and potentially GDM as well. Several of these IRS kinases (e.g., S6K1, PKC) are both inducers of insulin resistance and activated in response to insulin. The insulin-signaling pathway is highly conserved in all organs that express insulin receptors, including the brain [33,35]. In the brain, insulin receptors are expressed in the hippocampus region, which is responsible for memory and cognition [36]; down-regulation of IR in the hippocampus has been reported to decrease learning abilities and induce memory impairment in rats, as evaluated in a Morris Water Maze test [32]. Furthermore, this study also discovered that down-regulation of IR in the hippocampus inhibits the activation of glutamatergic system via decreasing phosphorylation of GluA1 and down-regulation of GluN2B [32]. The glutamatergic system is responsible for regulating synaptic plasticity and hence hippocampus-dependent spatial learning. The impairment of insulin signaling in the brain may also cause the activation of GSκ3β, where, under normal circumstances, it is inactivated when PI3K/AKT pathway is triggered as a result of insulin binding to IR. GSκ3β causes the phosphorylation of τ protein, consequently blocking the binding of the τ protein to microtubules. This results in the τ protein becoming unstable and will then disintegrate [34]. The unbound τ accumulates together and forms neurofibrillary tangles (NFT) [78,79,80]. Elevation of NFT will cause neuronal loss, which is associated with cognitive defects [34]. GSκ3β also releases pro-inflammatory cytokines (IL-2, IL-6 and TNF-α), which have neurotoxic effects on the CNS (see later section).

Impairment of the insulin-signaling pathway can also lead to the production of Aβ protein, which is derived from the amyloid precursor protein (APP) from sequential cleavage by β and γ secretase. Under normal conditions, APP is cleaved by α-secretase, where soluble APP fragments (sAPPα) are released into the extracellular space and partake in synaptic plasticity and cell survival. However, in memory-related diseases such as AD and dementia, APP is predominantly cleaved by β and γ secretase, induced by oxidative stress and hence produces a high concentration of Aβ. Studies by Solano et al. [81] and Gasparini et al. [82] showed that insulin treatment increased sAPPα secretion in a concentration and tyrosine kinase-dependent manner in neurons, reducing Aβ level. This suggests that sAPPα production is mediated by insulin [83] through the activation of insulin-signaling pathway [84,85]. Talbot et al. [33], in a case of cohorts from the University of Pennsylvania and the Religious Order Study, discovered a correlation between serine-phosphorylated IRS-1 and Aβ plaques, where serine-phosphorylated IRS-1 was found to be positively correlated with oligomeric Aβ plaques and negatively correlated with working and episodic memory. Besides PI3K/AKT, several other members of the insulin-signaling pathway have been studied for their involvement in cognitive dysfunction. Su et al. [86] compared the expression of 20 genes of the insulin-signaling pathway between 87 MCI subjects and 135 controls. Cognitive performance was also analyzed over a period of 35 months and it was found that 8 out of the 20 genes could be associated with cognitive performance. AKT1 and ATK2 were related to information processing speed and general cognition, respectively. Episodic memory was influenced by IGF1R and PIK3CB, and executive functioning was influenced by insulin degrading enzyme (IDE), PIK3CD, mTOR and AKT1S1 [86].

### 2.3. Oxidative Stress

Pregnant women experience an increase in oxygen consumption due to their increase in energy expenditure [87]. In GDM, oxidative stress, an imbalance between reactive oxygen species (ROS) and anti-oxidant enzyme levels was observed [88,89,90]. This has prompted the suggestion that oxidative stress contributes to the pathophysiology of GDM although concrete links have yet to be established; however, the link between T2DM and oxidative stress has been strongly established [91,92,93]. Alteration in free radical–anti-oxidant balance, glucose oxidation, lipid peroxidation, and production of AGEs, which are common in diabetes mellitus, can result in elevated levels of ROS, causing a state of oxidative stress. In pregnancy, a higher-than-usual level of ROS can be attributed to increased amounts of transitional metal ions e.g., iron and a mitochondria-rich placenta [91]. ROS modulates the insulin-signaling pathway via two mechanisms. The first mechanism involves the production of ROS in response to insulin where higher organisms have evolved the use of NO and ROS as signaling molecules for other physiological functions including regulation of vascular tone, monitoring of oxygen tension in the control of ventilation and signaling transduction from membrane receptors in various physiological processes. In the second mechanism, ROS negatively regulates the insulin pathway leading to reduced insulin secretion and consequently insulin resistance [37]. ROS trigger insulin resistance in the peripheral tissues by affecting various points in insulin receptor signal transduction, ultimately resulting in decreased expression of GLUT4 transporter in the cellular membrane [94]. Besides increased level of ROS, anti-oxidant enzymes such as SOD and CAT were found to be lower in diabetic control rats [43,44,45]. Superoxide dismutase activity and protein carbonyl content in the placenta were also found to be relatively low in GDM [95]. Superoxide dismutase regulates superoxides, which is a by-product of glucose metabolism and causes cellular damage.

The brain is an organ that is very sensitive to oxidative insults due to its high oxygen consumption rate, abundant lipid content and low anti-oxidant enzymes [96]. Oxidative stress in the brain may promote apoptosis, cell damage, disruption of neuronal function, and loss of synapse, all of which leads to cognitive dysfunction [47]. As previously mentioned, oxidative stress in the brain also promotes the cleavage of APP by β and γ secretase, producing a high concentration of Aβ. Brain insulin resistance may further produce high free radicals and reduce anti-oxidant enzymes in the brain that are already low in GDM to begin with. Free radicals can cause chemical modifications of proteins, lipids, DNA and RNA, thereby compromising the functional and structural integrity of neurons [48]. Consequently, loss of membrane cell functions and reduced neurotransmitter function and neuronal plasticity may be observed. The increase in free radicals also activates the NF-κβ pathway [41] that generates cytokines, where its role in GDM/cognition will be explored more in the next section.

One of the ways brain insulin resistance can increase free radical production is through inducing mitochondrial dysfunction (see Figure 1). It has been suggested that cognitive dysfunction can be attributed to mitochondrial dysfunction, which highly exposes the brain to apoptosis [97], a mechanism of cell loss in neurodegenerative diseases [97]. Mitochondria, an important organelle that converts energy into ATP but it is also a site of free radical production [47]. In the mitochondria, free radicals are produced mainly by Complex I (NADH CoQ reductase) and Complex III (bc1 complex) [98]. Insulin regulates mitochondrial function through the activation of the PI3K/AKT pathway, inhibiting the transcriptional factor, FOXO1. FOXO1 is hyperactivated in insulin resistance, inducing HMOX1, which oxidizes heme to biliverdin and ferric ion (Fe^3+^) [99]. Heme is important in both the function and stability of electron transport proteins. The depletion of heme roots the dysfunction of the mitochondrial electron transport chain, hence resulting in mitochondrial dysfunction [100] leading to an increased free radical production and decreased ATP production [47]. Brain insulin resistance can also lead to the reduction of anti-oxidant enzymes, by increasing the expression of p53 via inactivation of PI3K/AKT signaling cascade. Pro-apoptosis genes p53, NOS and NOX, which will result in reduced expression of genes encoding mitochondria-encoded complexes IV and V, function in the frontal lobe [38]. Undoubtedly, abnormalities in mitochondrial complexes, especially the activity of complex IV, which is the most documented reduction of mitochondrial enzyme activity that plays a vital role in cell degeneration and death, is seen in cognitive dysfunctions [49,50,51,52]. A study by Correia et al. [101] showed that rats administered with streptozotocin (STZ)-through intracerebroventricular (icv) injection experienced brain insulin resistance in addition to mitochondrial abnormalities with increased free radical levels. As stated earlier, increased production of free radicals in the brain may cause apoptosis and necrosis where it can inhibit neurogenesis [102]. Elevated levels of p53 gene activate nitric oxide synthase isoforms, mainly NOS 1–3 and NADPH-oxidase (NOX1 and 3) [38]. This inhibits anti-oxidant enzymes such as SOD, CAT, and GPX [39,40,41,42]. Hyperglycemia can also reduce the anti-oxidant enzyme level, particularly glutathione [103], produced from the reduction of glutathione disulfide (GSSG) by glutathione reductase enzyme and NADPH as a cofactor [104]. NADPH is also a cofactor for also aldose reductase, which converts glucose to sorbitol, and subsequently gets converted to fructose by sorbitol dehydrogenase in the polyol pathway [105]. In diabetic conditions, activation of the polyol pathway increases in parallel with glucose level, causing a decrease in NADPH and consequent decreases in GSH level [46].

### 2.4. Neuroinflammation

One of the characteristics of pregnancy is an altered inflammatory profile. It is crucial to have a highly regulated balance between pro- and anti-inflammatory cytokines in pregnancy, particularly for normal implantation, trophoblast invasion, and placentation [106]. However, in cases of GDM, a pro-inflammatory state has been reported [107,108,109,110]. In addition, the inflammation experienced in GDM is metabolically induced; hence the term metainflammation was coined, which leads to insulin resistance [111]. Furthermore, metainflammation displays these characteristics: (i) linked to a low metabolic rate; (ii) low-grade response; (iii) immune cells in pancreas, adipose tissue, and liver are altered to favor a pro-inflammatory state; and (iv) maintained by metabolic cells e.g., adipocytes [106]. As previously mentioned, adipose tissue release adipocytokines that induce insulin resistance and adipocytokines such as TNF-α, IL-1β, and IL-6 are pro-inflammatory cytokines, promoting a state of chronic low-grade inflammation [53,56]. Adipocytokines also releases leptin and adipokines, where leptin increases insulin sensitivity via increased insulin secretion, glucose use, glycogen synthesis, and fatty acid metabolism. Prospective studies have shown that GDM is linked to the down-regulation of anti-inflammatory cytokines (e.g., IL-4 and IL-10) and up-regulation of pro-inflammatory cytokines implicated in insulin resistance (e.g., IL-6 and TNF-α) [53]. Hyperglycemia and insulin resistance can also trigger the release of these cytokines where the former is through the formation of AGEs and the latter is through the inactivation of the PI3K/AKT signaling cascade, which activates GSK3β that releases pro-inflammatory cytokines. Neuroinflammation can lead to the development of cognitive impairment, and several studies have reported the presence of inflammation in the brains of cognitive dysfunction [62,112,113]. Another possible way that inflammation leads to cognitive decline is through the activation of microglia [55,59]. Activated microglia proliferates into macrophages, a main form of active immune defense in the CNS [60,61]. Pro-inflammatory cytokines such as IL-2, IL-6 and TNF-α are neurotoxic substances that can stimulate microglia activation (see Figure 1) [55]. Activation of microglia can cause alterations in the gene expression of various neurotoxic mediators such as TNFα, IL-1β, nitric oxide, superoxide, eicosanoids, and quinolinic acid [60,114,115]. This will attract leukocytes to the CNS, which amplifies the cycle of inflammation, inducing apoptosis and vascular breakdown, promoting glial impairment and neuronal cell death [55,116]. As explained previously, oxidative stress induces production of Aβ, which in turn activates the NF-κβ via binding of Aβ with RAGE [117,118]. NF-κβ is identified as one of the main regulator of inflammation and oxidative stress [55] that controls the expression of several important genes involved in the immune response. Activation of NF-κβ results in the release of pro-inflammatory cytokines such as TNF-α and interleukins (IL-2, IL-6, IL-8) [55]. The hippocampus region of brains derived from STZ-rats showed increased expression of NF-κβ resulted in an elevated level of pro-inflammatory cytokines [56]. In a study of 276 diabetic elderly by Gorska-Ciebiada et al. [112] several inflammatory markers e.g., TNF-α, CRP and IL-6 and its correlation with MCI, assessed through the Montreal Cognitive Assessment. It was found that the MCI groups showed a higher level of the three inflammatory markers compared to control; however, the difference between the two groups regarding IL-6 was not statistically significant [112]. However serum levels of IL-1β and leptin were higher in MCI group compared to control [119]. In normal pregnancy, leptin is usually high in early until late pregnancy [120] and will return to normal after delivery. In GDM, level of leptin is higher than in normal pregnancy and remains high after pregnancy [57]. This may be due to the high production of inflammatory cytokines such as TNF-α and IL-6 [121,122], thus further amplifying inflammatory conditions. In normal pregnancy, adiponectin level is low in the first trimester, but the level further drops in the third trimester when insulin resistance is at its peak. However, in GDM, the level of adiponectin is significantly lower than in normal pregnancy. Adiponectin exhibits an anti-inflammatory effect by inhibiting phagocytic activity of macrophages that produces TNF-α [123]. However, inflammatory mediators such as TNF-α can also block the production of adiponectin. Inflammation can also affect cerebral vasoregulation, which could accelerate the advancement of cognitive decline in diabetic cases [54,62]. Chung et al. [54] reported that a high level of vascular adhesion molecules and hs-CRP can be correlated with decreased cerebral vasodilation and vasoreactivity. Furthermore, the decrease in vasoreactivity was positively correlated with a decline in multiple cognitive tasks in diabetic patients. Peroxisome proliferator-activated receptor γ (PPAR-γ), a nuclear receptor subfamily, that causes insulin sensitization and enhances glucose metabolism [124] may be implicated in cognitive impairment in GDM. PPAR-γ mRNA level was lower by 38% in pregnant control and 48% in GDM subjects compared with non-pregnant controls [58]. More importantly, PPAR-γ also has anti-inflammatory activity where it blocks NF-κβ and reduces TNF-α level [125], and several studies have shown the neuroprotective effect of PPAR-γ agonists in animal models [63,64]. Decreased PPAR-γ and its target genes may be part of the molecular mechanism to accelerate fat catabolism to meet fetal nutrient demand in late gestation [58].

## 3. Future Directions and Conclusions

This review focuses on cognitive impairment in GDM and the different pathologies of GDM that can lead to cognitive impairment. The pathologies outlined are also suggested to be involved in T2DM-related cognitive decline; hence it is reasonable to consider the shared pathologies as possible contributors to their complications as well. Here, we would like to highlight several notes that can aid future research in GDM-associated maternal cognitive impairment:

(a) Potential biomarkers and molecules for cognitive decline includes AGEs, serine-phosphorylated IRS-1, and cytokines such as TNF-α, hs-CRP, leptin, IL-1β, and IL-6. The level of these molecules should be evaluated in GDM cases in the future and in relation to cognitive decline.
(b) The PI3K and AKT signalling cascade is important in the downstream insulin pathway in GDM. Moreover, there is also crosstalk with other pathways that are important in maintaining cognitive function through increasing or decreasing key regulators of cognitive function such as GSK3β.

However, one should also be cautious in relating complications seen in T2DM with GDM. Although the similarities between both do provide some clues when investigating GDM-related cognitive impairment, there are some differences between T2DM and GDM that need to be taken into account. Firstly, pregnancy hormones play a pivotal role in the pathophysiology of GDM and therefore its involvement in GDM-associated maternal cognitive impairment should be explored as well. Secondly, GDM is a transient disease where, in most cases, blood sugar level will return to normal after delivery. This provides a major setback in GDM research where it is difficult to determine whether complications related to GDM and its effects are long-term. Thirdly, while the diseases may share similarities on a genotypic and phenotypic level, they have different risk factors, such as age and gender, which may play contributing role in their pathophysiologies. 

There is clearly a need for more research in understanding cognitive impairment in GDM even though GDM-related pathologies such as hyperglycemia, insulin resistance, oxidative stress, and neuroinflammation have been extensively explored in the cognitive impairment of different diseases e.g., AD and T2DM. As new knowledge is gained, it can be applied to develop new and improved ways to prevent and treat GDM and its related complications.

## Figures and Tables

**Table 1:** Summary of the proposed pathophysiological alterations in maternal cognitive impairment associated with GDM.

Evidence	Markers	Pathophysiological Changes	Adverse Effects and Outcome
Hyperglycemia	Advanced glycation end products (AGEs) [26].	Dysfunction of neurovascular unit and regulation of cerebral blood flow [27,28]. Integrity of the blood-brain-barrier (BBB) and its transport functions affected [27]. Abnormal proliferation of endothelial cells, thickening of capillary walls, low perfusion rates and increased vascular permeability [27].	Vascular damage of the BBB hinders the transport of choline, Reduced clearance of amyloid-β (Aβ) protein promotes tau (τ) phosphorylation [29,30]. Induces oxidative stress and inflammation
Insulin resistance	GLUT 4 [31], GluA1, GluN2B [32], IRS-1 [33], GSK3β [34].	Brain insulin resistance is a direct result of peripheral insulin resistance by limiting transport of insulin into the CNS [33,34,35].	Down-regulation of IR in the hippocampus inhibits the activation of glutamatergic system [32,36] resulting in low learning abilities, reduced synaptic plasticity and hippocampus-dependent spatial learning [32]. Low brain insulin cause hyperphosphorylation of τ protein and/or high expression of Aβ protein [34].
Oxidative stress	ROS [37], p53 [38], NOS/NOX, [38,39,40,41,42] SOD, CAT, GPX [43,44,45], GSH [46].	Increased free radicals promote apoptosis, cell damage, disruption of neuronal function and loss of synapse [47]. ROS causes insulin resistance [37]. Loss of membrane cell functions and reduced neurotransmitter function and neuronal plasticity [48].	ROS promotes the cleavage of APP by β and γ secretase, producing high concentration of Aβ [47]. Free radicals cause chemical modifications of proteins, lipids, DNA and RNA, thereby compromising neuronal functions [48]. Abnormalities in mitochondrial complexes reduce mitochondrial enzyme activity that is vital in neuronal degeneration and death [47,49,50,51,52].
Neuro- inflammation	IL-6 and TNF-α [53], IL-2, hs-CRP [54], IL-8,NF-κβ [55] IL-1β, [56], Leptin [57], PPAR-γ [58].	Activation of microglia [55,59] and proliferation into macrophages in the CNS [60,61]. Long cycle of inflammation induces apoptosis and vascular breakdown, promoting glial impairment and neuronal cell death [55]. Inflammation affect cerebral vasoregulation [54,62]. Decreased PPARγ and its target genes [58].	Activation of microglia causes alterations in the gene expression of various neurotoxic mediators [55]. Neuronal damage [63,64].
